# The prevalence of Restless Legs Syndrome/Willis-ekbom disease (RLS/WED) in the third trimester of pregnancy: a systematic review

**DOI:** 10.1186/s12883-020-01709-0

**Published:** 2020-04-13

**Authors:** Niloofar Darvishi, Alireza Daneshkhah, Behnam Khaledi-Paveh, Aliakbar Vaisi-Raygani, Masoud Mohammadi, Nader Salari, Fateme Darvishi, Alireza Abdi, Rostam Jalali

**Affiliations:** 1 Department of Nursing, School of Nursing and Midwifery, Kermanshah University of Medical Sciences, Kermanshah, Iran; 2 School of Computing, Electronics and Maths, Coventry University, London, UK; 3 Sleep Disorders Research Center, Kermanshah University of Medical Sciences, Kermanshah, Iran; 4 Department of Biostatistics, School of Health, Kermanshah University of Medical Sciences, Kermanshah, Iran; 5 School of Medicine, Kermanshah University of Medical Sciences, Kermanshah, Iran

**Keywords:** Prevalence, Restless legs syndrome, Pregnancy, Systematic review

## Abstract

**Background:**

RLS is known as one of the most common movement disorders during pregnancy, which is most aggravated in the third trimester of pregnancy and can affect up to one-third of pregnant women. This study intends to determine the total prevalence of RLS in the third trimester of pregnancy through a systematic review.

**Methods:**

The present study was conducted via meta-analysis method up to 2019. The papers related to the subject of interest were obtained through searching in SID, MagIran, IranDoc, Scopus, Embase, Web of Science (ISI), PubMed, Science Direct, and Google Scholar databases. Heterogeneity of the studies was examined via I^2^ index, and the data were analyzed in Comprehensive meta-analysis software.

**Results:**

In investigating 10 papers capturing 2431 subjects within the age range of 25–39 years, the total prevalence of RLS in the third trimester of pregnancy based on meta-analysis was obtained as 22.9% (95% CI: 14.7–33.8%). Further, as the sample size increased, the RLS prevalence diminished, while with increase in years, this prevalence increased, where this difference was statistically significant (*P* < 0.05).

**Conclusion:**

Prevalence of RLS in the third trimester of pregnancy is high, healthcare policymakers should organize educational classes to improve the life dimensions among this group of pregnant women.

## Background

Sleep is a very important physiological need for everyone, which is absolutely essential for the physical and physiological health [1]. Sleep disorders are among the most important issues in medical care. Sleep disorders are generally categorized as disturbed quality, poor sleep continuity, Restless Legss Syndrome, sleep disorder, and sleep respiratory disorder [2]. Pregnancy is one of the most important periods in the life of women. This period is associated with major physiological, psychological, and social changes [3]. Around two-thirds of pregnant women consider their sleep patterns as abnormal [3]. The relationship between sleep disorders and pregnancy complications is biologically acceptable [4].

Poor health outcomes because of the biological and secretion modification throughout pregnancy might even be associated with sleep disturbances [2].

The prevalence of RLS is 5 and 15% globally and among Caucasians, respectively [5]. RLS affects up to one-third of pregnant women, which peaks in the third trimester of pregnancy, and then usually declines after the delivery [6].

The results show that the prevalence of Restless Legs Syndrome/Willis-Ekbom is associated with pregnancy months, with the highest prevalence in the third trimester, especially in the seventh and eighth months. Among those who suffered from this disorder before pregnancy, 11% showed improvement in symptoms during pregnancy, 28% did not notice any change, and in 5% the symptoms worsened. Postpartum, the prevalence of Restless Legs Syndrome/Willis-Ekbom also decreased, with fewer women reporting symptoms of 6.8% a month postpartum [7].

As results of a study in Saudi Arabia revealed, the prevalence of RLS in the third, second and first trimester was 24.1, 14.3 and 13.6%, accordingly [8]. Similar article showed 34.3, 35.3 and 30.4% of RLS prevalence in the third, second and first trimester, respectively [9]. But the prevalence of RLS in another study was reported 38.8 in the third trimester, 32.8% in the second trimester, and 15.6% in the first trimester [10].

The reason behind development or aggravation of RLS during pregnancy is still unclear, for which several hypotheses have been propounded so far [11–13]. Endocrine changes, iron and folate metabolism, genetics, and other factors are among these hypotheses. Endocrine changes: during pregnancy, many endocrine changes occur; Estradiol, progesterone, and prolactin rise. All of these elevations occur in particular in the third trimester, which may explain induction of RLS. Iron and folate metabolism: during pregnancy, the level of serum iron, ferritin, and folate diminishes, possibly due to their dilutions in a larger blood volume, given the demand by the fetus. Genetics: there is a hypothesis suggesting that pregnancy may cause initiation of symptoms in women who are genetically predisposed to developing RLS [14–18].

A number of other possible causal factors of RLS during pregnancy has been investigated. These factors include comorbidity with other sleep disturbances, such as respiratory disorders; RLS and excessive daytime sleepiness; psychological conditions exacerbating RLS symptoms, such as anxiety, stress, and lower limb hypoxia or edema [14].

In order to treat RLS, in mild cases, typically nonpharmacological methods such as stretching the legs before sleep and use of elastic socks when accompanied by varicose veins are suggested [14–18]. If RLS symptoms are mild or trivial, changes in the lifestyle such as regular exercise may be beneficial. Massaging the legs or soaking them in water may also be alleviating [19]. However, when RLS is severe, pharmacotherapy may be indicated. Pharmacotherapy should be used based on the age of the patient and comorbidities in a step-by-step fashion [20–24]. The medications prescribed for RLS include pramipexol, ropinirole, Rotigotine patch, and levodopa [25–28].

Since RLS is known as one of the most common movement disorders during pregnancy which peaks during the third trimester and affects up to one-third of pregnant women, this study aims to determine the total prevalence of RLS in the third trimester of pregnancy through a systematic review.

## Methods

### The search method

This study has been conducted as systematic review. It is an outcome of extracting the findings of the studies on RLS prevalence in the third trimester of pregnancy, which include papers published in both domestic and foreign journals as well as search in SID, MagIran, IranDoc, Scopus, Embase, Web of Science (ISI), PubMed, Science Direct, and Google Scholar databases up to 2019. The search process in these databases proceeded through the keywords of restless legs syndrome, pregnancy, third trimester of pregnancy both in English and Persian as well as their combinations. The search performed in Google scholar search engine was performed based on both English and Persian keywords. In this investigation, (AND) and (OR) operators were used for combining the keywords for comprehensive and complete access to papers.

After concealing the specifications of papers including the name of the journal and author, the full text of the papers was provided to reviewers. Every paper was studied by two reviewers independently; in case the paper was rejected, its reason of rejection was mentioned and if any disagreement existed between the two reviewers, it would be judged by a third reviewer.

### The criteria for selection and assessment of papers

**Fig. 1:**
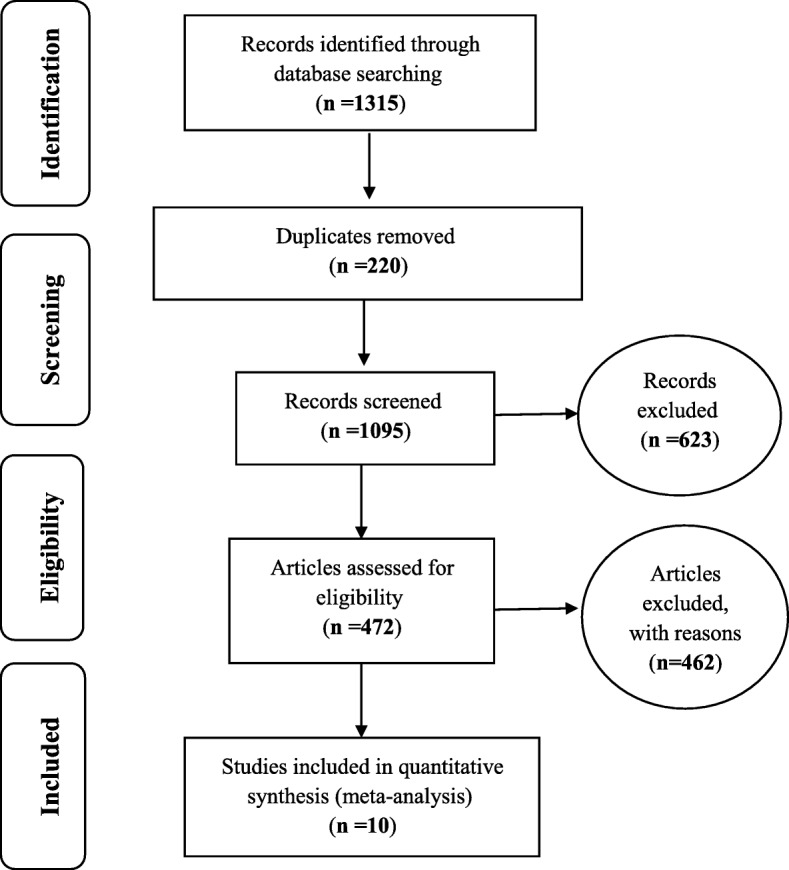
The flowchart on the stages of including the studies in the systematic review and meta-analysis (PRISMA 2009)

The Persian and English papers adapted from cross-sectional studies on RLS during the third trimester of pregnancy met the inclusion criteria to be included in the paper. On the other hand, other review studies, observational, and interventional studies were excluded from the list of the papers. Thereafter, the included studies were investigated based on PRISMA 2009 (Fig. 1). In order to examine the studies, STROBE checklist was employed. Accordingly, the maximum quality check score was considered as 32.

### Statistical analysis

Data analyzed by Comprehensive Meta-analysis software (Biostat, Englewood, NJ, USA, Version 3). The heterogeneity of studies was examined by I^2^ test and the probability of publication bias of the results was measured by funnel plot using Egger test.

## Results

### Publication bias

**Fig. 2:**
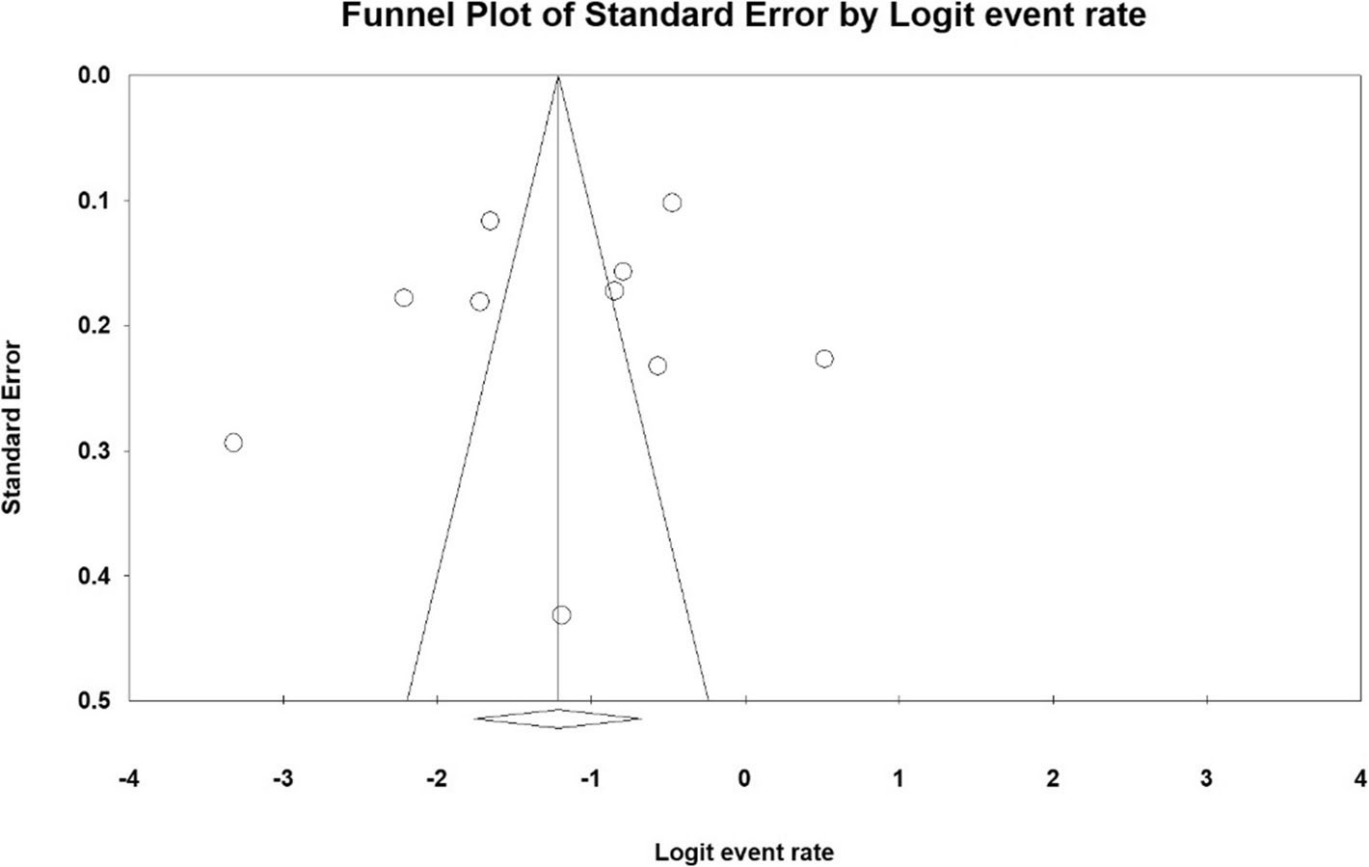
The funnel plot of the results related to RLS prevalence in the third trimester of pregnancy

The results obtained from investigation of publication bias across the studies were measured by Egger test (Fig. 2), which indicated that the bias was not statistically significant (*P* = 0.579).

Based on studies on the prevalence of RLS in third trimester of pregnancy that included articles published either in domestic or foreign journals, a total of 1315 articles were obtained; then articles that met the initial inclusion criteria were included in the study but via primary assessment, the exclusion of 220 duplicate ones left 1095 articles that by elimination of another 623 articles unrelated to subject of study and the elimination of 472 articles in a secondary assessment due to the lack of access to the abstract and full text of the articles and because of the poor quality of the articles eventually 10 articles entered the meta-analysis process (Table 1).

**Table 1:** Specifications of studies entered the study

Row	Author [References]	Publication year	Area	Participants’ Age	RLS scale	RLS Questionnaire scale	Sample size	Prevalence
1	Facco, F. L. [4]	2010	America	29.7 ± 5.5	International Restless Legs Syndrome Question Set	self-administered questionnaire	189	31.3%
2	Neau [20]	2009	France	29.2	International RLS (IRLS) Study Group	self-administered close-ended questionnaire	355	21.7%
3	Sarberg [21]	2012	Sweden	30.1	International RLS Study Group	After written and oral information	160	29.60%
4	Terzi [22]	2015	Turkey	28.0 ± 5.5	International RLS Study Group	Face to face interview questionnaire	83	62.7%
5	Alves [23]	2010	Brazil	27.3	International RLS study group rating scale (IRLS) and IRLSSG in 2003	clinical-diagnostic interview	237	15.18%
6	Harano [24]	2008	Japan	29.9 ± 4.6	International RLS Study Group	selfadministrated questionnaire	344	3.5%
7	A.lee [25]	2001	California	25–39			30	23%
8	Neau [26]	2010	France	28.8 ± 4.6	International RLS (IRLS) Study Group	self-administered close-ended questionnaire	406	38.4%
9	Shang [27]	2014	China	26.0 ± 6.4	International Restless Legs Syndrome Study Group (IRLSSG)	Face to face interview	547	16.1%
10	Shahzad [28]	2018	Pakistan (Lahour)	28.2 ± 5.4	International RLS study group rating scale	interviewed via well-defined pre tested questionnaire	80	36.3%

### Examination of the heterogeneity and meta-analysis

**Fig. 3:**
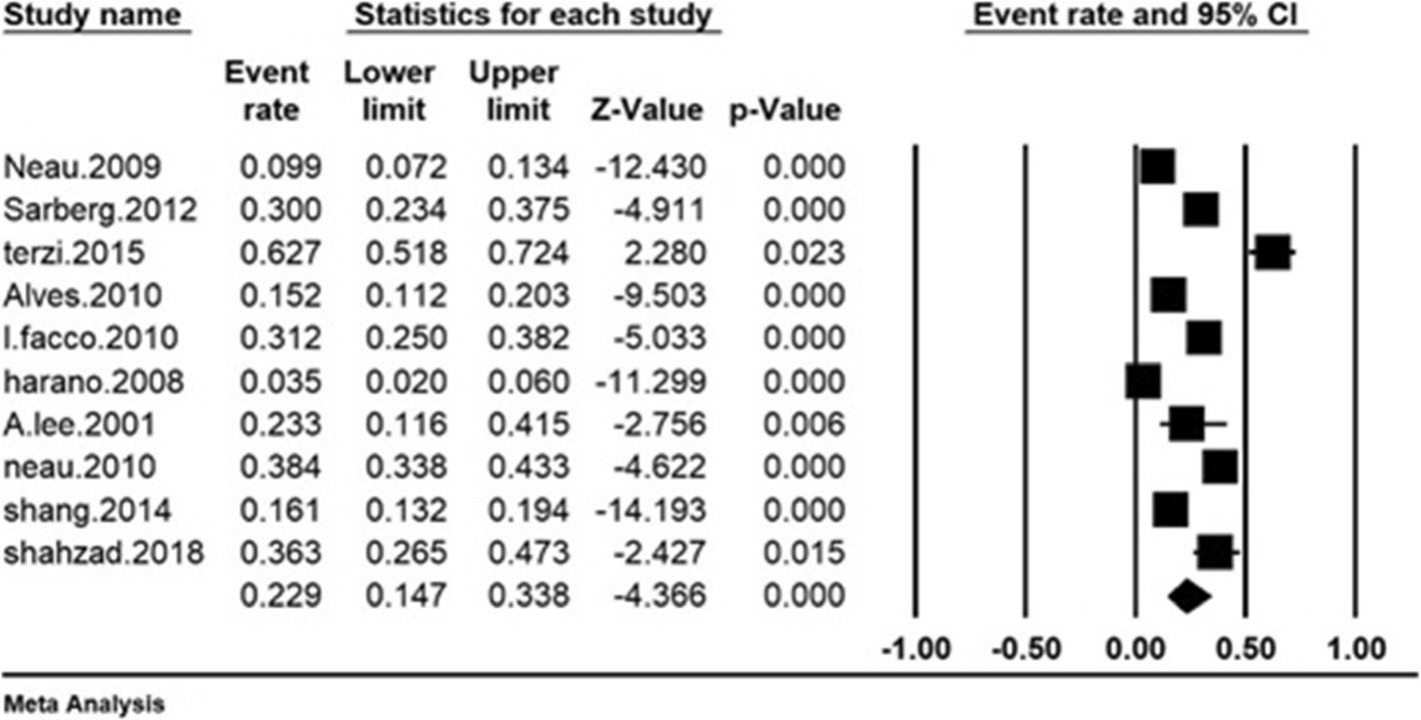
Total prevalence of RLS in the third trimester of pregnancy based on the random model (the black square represents the prevalence rate and length of line the square is situated on shows the 95% confidence interval in each study. The diamond symbol shows the prevalence rate in the whole country for the total studies)

Based on which I^2^ = 96% was obtained, suggesting high heterogeneity across the included studies. Thus, random effects model was utilized to combine the results of the studies together. The total number of participants in the study was 2431 individuals aged between 25 and 39 years old, The overall prevalence of RLS in the third trimester was 22.9% (95% CI: 14.7–33.8%) based on meta-analysis the highest prevalence of restless legss syndrome in third trimester was in the study of Terzi et al. [22] in Turkey (62.7%) and the lowest prevalence of RLS in third trimester was in the study of Harano et al. [24] in Japan (3.5%) (Fig. 3).

**Fig. 4:**
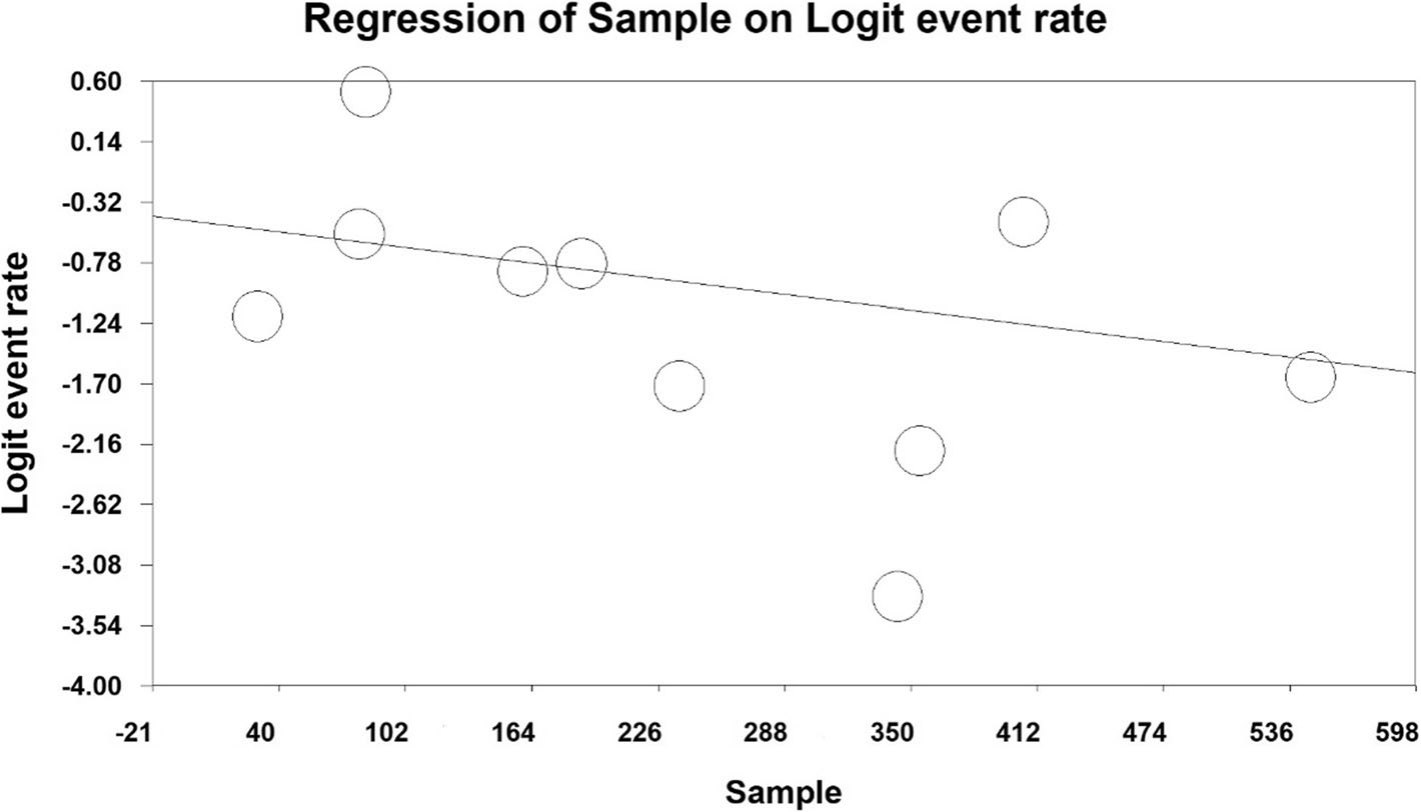
The meta-regression diagram of RLS prevalence in the third trimester of pregnancy per sample size

**Fig. 5:**
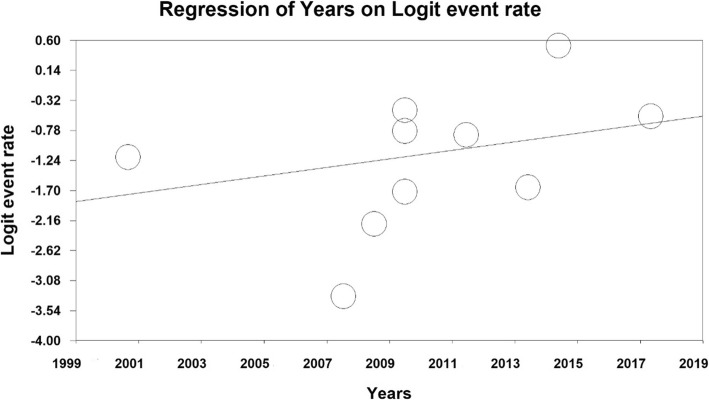
The meta-regression diagram of RLS prevalence in the third trimester of pregnancy per year of conducting research

According to the meta-regression in Figs. 4 and 5 reported, with increase in the sample size, RLS prevalence has decreased, which is statistically significant (*p* < 0.05) (Fig. 4), also reported that RLS prevalence was higher in more lately published papers, which was a statistically significant (*p* < 0.05) (Fig. 5).

## Discussion

The aim of this study was to determine the prevalence of restless legs syndrome in the third trimester of pregnancy. Our systematic review of published epidemiologic studies confirmed a 22.9% prevalence of RLS in the third trimester of pregnancy. To the best of our knowledge, it was the first meta-analysis of prevalence of RLS in late pregnancy. In total, we have used 10 studies in this meta-analysis to address the purposes of this study.

Pregnancy is one of the most important underlying factors for sleep disorders [29]. Restless legs syndrome can occur after pregnancy. RLS occurs more frequently in pregnant women as compared to the general population [30].

According to a study by Khan, M. et al. in Saudi Arabia, the prevalence of RLS in pregnancy was reported to be 21.3%, which was specifically observed in the third trimester of 24.1% [8].

Another study reported that the prevalence of restless legs syndrome was higher in the second and third trimesters of pregnancy, which 38.1% in the third trimester, the study was also found that a family history of restless legs syndrome and increased gestational age increase the risk of RLS [10].

It was also found that diseases such as arthritis, anemia and thyroid disorders are more common in patients with RLS [9], in study by Neyal et al., was reported that a family history of restless legs syndrome and increased gestational age increases the risk of RLS, It was also reported that blood urea nitrogen levels, high transferrin levels, and low levels of ferritin, increase the risk of restless legs syndrome [10].

Another study in China showed a higher prevalence of RLS in the third trimester of pregnancy, which was reported to be 16.1% [9], In the Manconi study, and the prevalence of RLS in pregnancy was 26.6% [7].

Based on the study by Balendran et al., which was conducted as case-control on pregnant women population in which 211 had been included, RLS prevalence in both the sample and control group populations was reported as 22.5% [31].

Also, based on another study performed as cohort, RLS prevalence had been reported as 36% among pregnant women (in that study, 1518 individuals had been included) [11]. Similarly, in another study by Hubner et al. conducted as cohort on 501 subjects, overall 58 (12%) of pregnant women had RLS [32].

Considering the results of the present study and an examination of 2431 people with age range of 25–39, the overall prevalence of RLS in third trimester of pregnancy was 22.9% that is on the basis of meta-analysis. Moreover, the results obtained from meta-regression reported that the prevalence of RLS in third trimester of pregnancy decreases with increased sample size and increases as the number of years increases which were both statistically significant.

### Limitation

The most important limitation of the present study is inaccessibility to the full text of the articles, incomplete search, and recruitment differences (population-based included health insurance data or center-based (included hospital referrals) in studies review.

## Conclusion

Prevalence of RLS in the third trimester of pregnancy is high, healthcare policymakers should organize educational classes to improve the life dimensions among this group of pregnant women, health policy makers need to hold effective training classes to improve dimensions of living among this group of pregnant women.

## Data Availability

Datasets are available through the corresponding author upon reasonable request.

## Abbreviations

RLS: Restless legs Syndrome
WED: Willis-Ekbom
SID: Scientific Information Database
MESH: Medical Subject Headings
ISI: Web of Science
PRISMA: Preferred Reporting Items for Systematic Reviews and Meta-Analysis.
STROBE: Strengthening the Reporting of Observational Studies in Epidemiology for cross- sectional Study
